# The therapeutic potential of cell cycle targeting in multiple myeloma

**DOI:** 10.18632/oncotarget.18765

**Published:** 2017-06-28

**Authors:** Anke Maes, Eline Menu, Kim De Veirman, Ken Maes, Karin Vand erkerken, Elke De Bruyne

**Affiliations:** ^1^ Laboratory of Hematology and Immunology, Myeloma Center Brussels, Vrije Universiteit Brussel, Brussels, Belgium

**Keywords:** cell cycle deregulation, multiple myeloma, cyclin-dependent kinases, aurora kinases, anaphase promoting complex/cyclosome

## Abstract

Proper cell cycle progression through the interphase and mitosis is regulated by coordinated activation of important cell cycle proteins (including cyclin-dependent kinases and mitotic kinases) and several checkpoint pathways. Aberrant activity of these cell cycle proteins and checkpoint pathways results in deregulation of cell cycle progression, which is one of the key hallmarks of cancer. Consequently, intensive research on targeting these cell cycle regulatory proteins identified several candidate small molecule inhibitors that are able to induce cell cycle arrest and even apoptosis in cancer cells. Importantly, several of these cell cycle regulatory proteins have also been proposed as therapeutic targets in the plasma cell malignancy multiple myeloma (MM). Despite the enormous progress in the treatment of MM the past 5 years, MM still remains most often incurable due to the development of drug resistance. Deregulated expression of the cyclins D is observed in virtually all myeloma patients, emphasizing the potential therapeutic interest of cyclin-dependent kinase inhibitors in MM. Furthermore, other targets have also been identified in MM, such as microtubules, kinesin motor proteins, aurora kinases, polo-like kinases and the anaphase promoting complex/cyclosome. This review will provide an overview of the cell cycle proteins and checkpoint pathways deregulated in MM and discuss the therapeutic potential of targeting proteins or protein complexes involved in cell cycle control in MM.

## INTRODUCTION

Multiple myeloma (MM) is a malignant plasma cell disorder characterized by the infiltration and accumulation of tumor cells in the bone marrow (BM), secretion of a monoclonal protein in the blood/urine and end organ damage [1]. MM arises from a premalignant asymptomatic stage, referred to as monoclonal gammopathy of undetermined significance (MGUS). This premalignant disorder progresses to smoldering myeloma (asymptomatic) and eventually to symptomatic MM with a rate of 0.5–1% per year [2, 3]. Symptomatic MM is defined by the presence of malignant plasma cells in the bone marrow (> 10% of all BM mononuclear cells) or biopsy proven plasmacytoma and one or more myeloma defining events. These myeloma defining events include evidence of end organ damage (including hypercalcemia, renal failure, anemia and bone lesions (CRAB symptoms)) or any of the following biomarkers of malignancy: clonal BM plasma cell percentage of 60% or more, involved/uninvolved serum free light chain ratio of 100 or more and more than 1 focal lesion on MRI studies [1, 3–5].

The malignant transformation from normal plasma cells to MM cells is established by the acquisition of multiple genetic abnormalities [4, 6, 7]. The initial genetic defects associated with MM development can be subdivided into the non-hyperdiploid and hyperdiploid group; both uniformly resulting in the deregulation of cyclin D genes [4]. The non-hyperdiploid defects are observed in around 45% of MGUS patients and 40–50% of all MM patients [4, 6, 8]. They include mainly translocations involving the immunoglobulin heavy chain (IgH) locus (14q32). The most frequent translocation is t(11;14)(q13;q32) and it results in an aberrant expression of the cyclin D1 gene. Other common translocations at the IgH locus involve t(4;14), t(14;16), t(6;14) and t(14;20), leading to the overexpression of cyclin D3, deregulation of the histon methyltransferase MMSET and upregulation of c-Maf and MafB, respectively. Both MMSET and Maf family deregulations result in the overexpression of cyclin D2 [4, 6]. In 50% of MGUS patients and up to 55% of MM patients hyperdiploidy is observed, specifically trisomies of chromosomes 3, 5, 7, 9, 11, 15, 19 and 21 [4, 6, 9]. Tumor progression is associated with secondary events such as Ras mutations, c-Myc overexpression, deletion of chromosome 13, constitutive activation of NF-κB, deletions of chromosome 1p and 17p, gain or amplification of chromosome 1q and inactivation of p53 [4, 7].

The growth and survival of these transformed plasma cells depends on the supportive conditions of the bone marrow microenvironment (BMM) [7, 10]. The BMM is composed of a cellular compartment (including hematopoietic cells, fibroblasts, osteoclasts, osteoblasts, adipocytes, endothelial cells, mesenchymal stem cells and immune cells) and an extracellular compartment consisting of growth factors, cytokines, chemokines and matrix metalloproteinases. The most potent growth factors for MM pathogenesis are vascular endothelial growth factor (VEGF), insulin-like growth factor 1 (IGF1) and interleukin 6 (IL6). The bidirectional communication between the MM cells and the BMM supports the growth, proliferation, adhesion and migration of the malignant cells and contributes to drug resistance. Moreover, it also results in immune suppression, increased angiogenesis and osteolysis [7, 10, 11].

MM is a complex malignancy to treat considering the high genetic heterogeneity and the critical role of the BMM in the pathogenesis of MM [12, 13]. Over the last decade, the introduction of drugs targeting MM cells in their microenvironment, such as immunomodulatory drugs (thalidomide, lenalidomide) and proteasome inhibitors (bortezomib, carfilzomib), has markedly improved the survival of MM patients. These so-called ‘novel agents’ are now standard of care agents and are used in combination with autologous stem cell transplantation and/or chemotherapy. However, almost all patients relapse or become refractory to these drugs. Therefore, other drugs are complemented to improve MM treatment in relapsed/refractory patients, such as agents targeting nuclear transport, histone deacetylase inhibitors and monoclonal antibodies [12–15]. Nevertheless, almost all MM patients will eventually relapse and become refractory to any treatment option, leaving MM most often an incurable disease [13, 15].

Deregulation of cyclin D genes is an uniform event in MM patients and results in an aberrant cell division [3, 16]. This emphasizes that the cell cycle is an interesting target in MM. Current chemotherapy used to treat myeloma includes the microtubule targeting agent (MTA) vincristine, which is an anti-mitotic drug. However, major limitations of this MTA are peripheral neuropathy (due to the critical role of microtubules in neuronal transport) and neutropenia (due to the toxic effect on hematopoietic progenitor cells) [17, 18]. Thus, new strategies that do not affect the microtubule are needed to improve anti-mitotic treatment. Therefore, current studies are focussed on more targeted strategies, such as cyclin-dependent kinase and aurora kinase inhibitors. This review will discuss the role of cell cycle deregulation in the pathogenesis of myeloma and focus on the therapeutic potential of targeting proteins or protein complexes involved in cell cycle as anti-myeloma therapy.

## REGULATION OF THE CELL CYCLE

### Cell cycle phases

**Figure 1:**
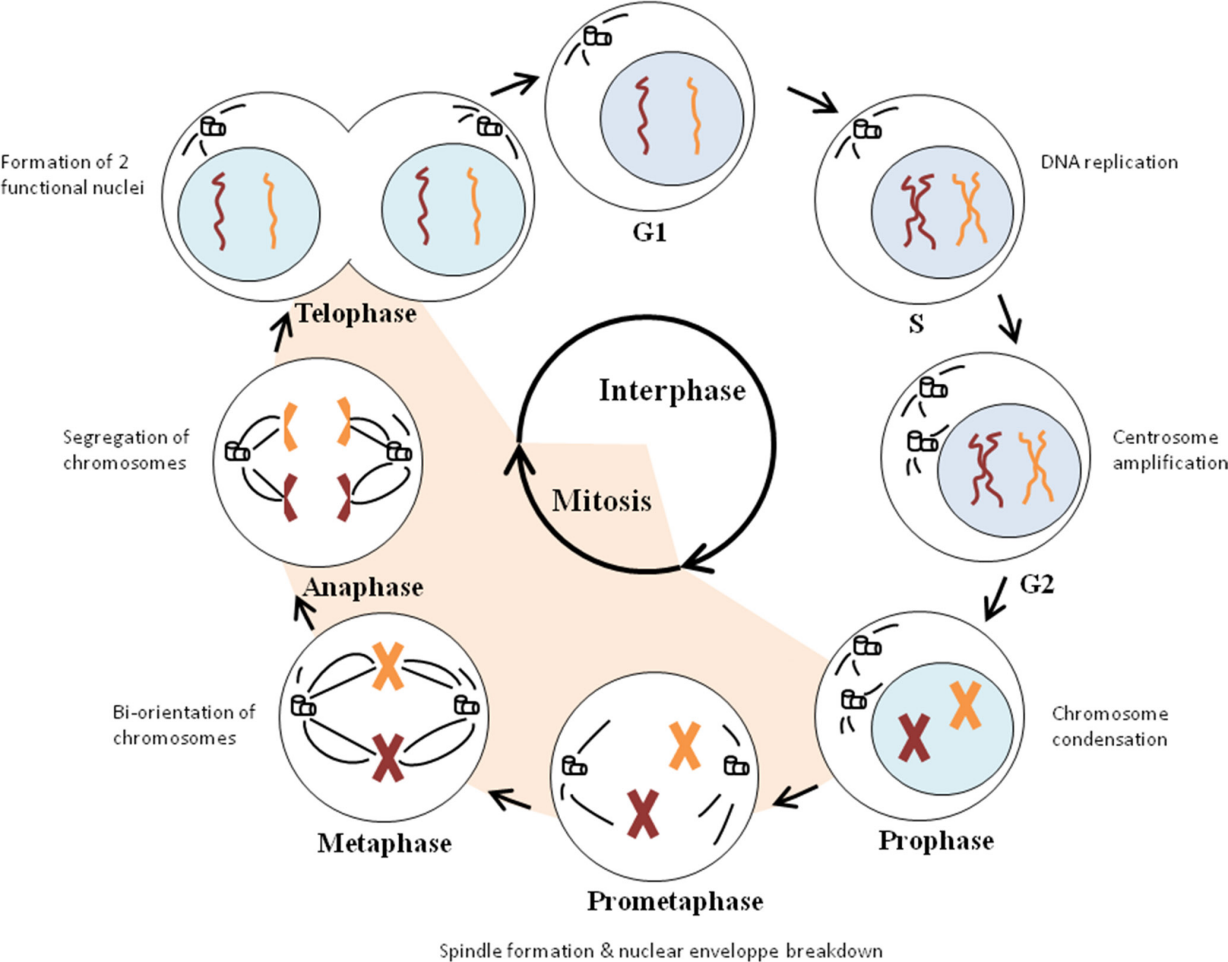
Cell cycle progression Cell division consists of an interphase and mitosis. The interphase (G1, S and G2 phases) is characterized by the growth in cell size (G1 and G2 phase), DNA replication (S phase) and centrosome amplification (G2 phase). The actual nuclear division occurs in mitosis (M-phase). Chromosome condensation takes place during the prophase, while spindle formation and nuclear envelope breakdown occurs in the prometaphase. In the metaphase, chromosomes achieve bi-orientation and the segregation of the sister chromatids takes place in the anaphase. During the last stage of mitosis, namely the telophase, two functional nuclei are formed and two daughter cells are produced.

The cell cycle is a process in which cells divide and reproduce themselves. This cell division consists of an interphase and mitosis, characterized by respectively DNA replication and nuclear division [19, 20]. The interphase includes the G1, S and G2 phases. During these phases, the cells grow in size. In the G1 phase, cells are preparing for DNA replication, which then occurs in S phase. However, before committing to DNA replication, cells can decide to leave the cell cycle and enter a resting state which is called the G0 phase. DNA synthesis is followed by a second gap phase (G2), in which the cells are preparing for nuclear division (mitosis). There are different stages during mitosis: the prophase, prometaphase, metaphase, anaphase and telophase. Events taking place in mitosis are chromosome condensation in prophase, spindle formation during prometaphase, attachment of chromosomes to the spindle and organisation of them in metaphase, segregation of chromosomes in anaphase and formation of two functional nuclei during telophase. Eventually two daughter cells are produced by cytokinesis (Figure 1) [20].

### Cell cycle regulation

**Figure 2:**
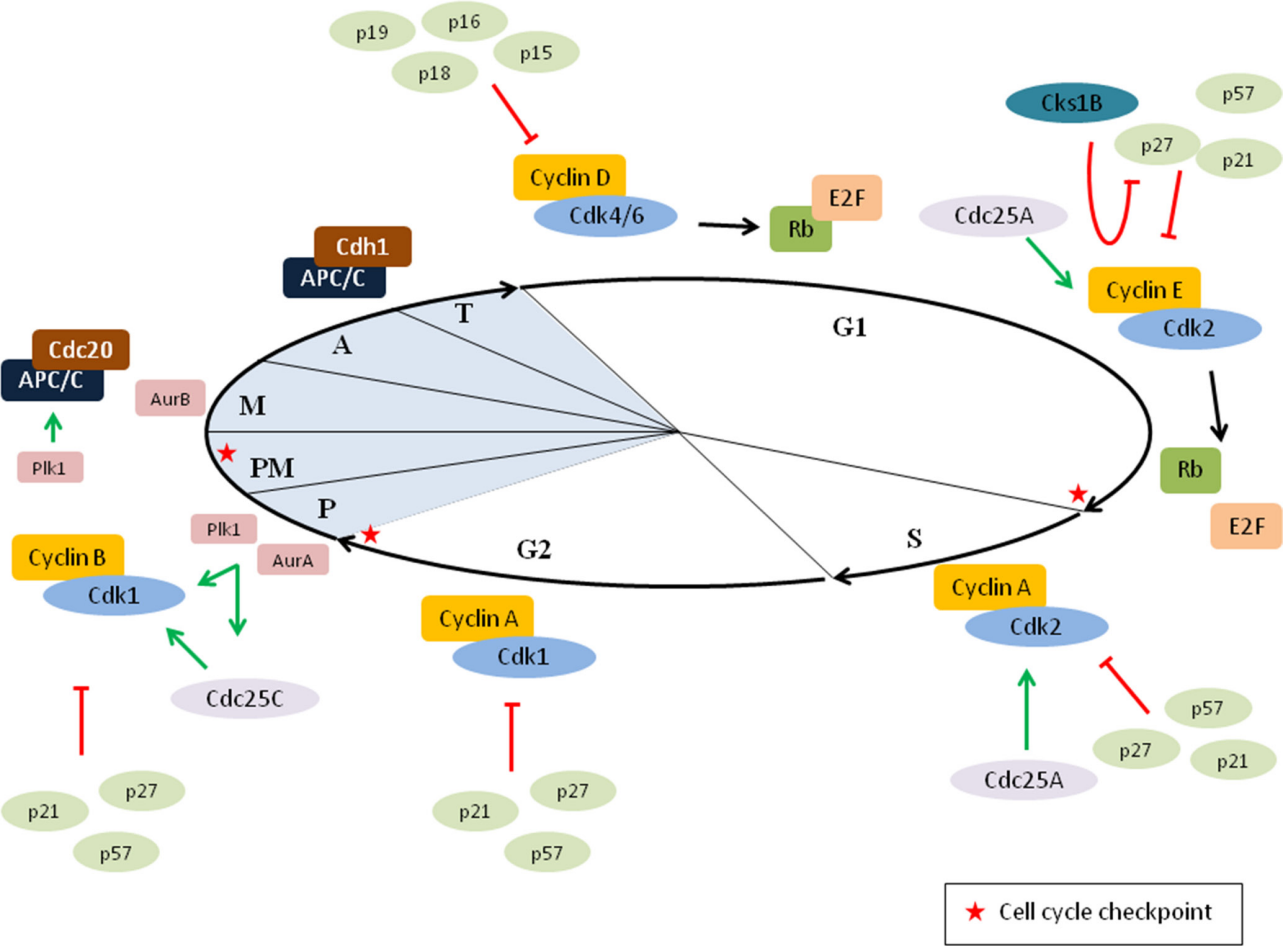
Cell cycle regulation Cell cycle progression is highly regulated by the coordinated activation of cyclin–cyclin-dependent kinases (Cdk) dimers. In early G1, cyclin D–Cdk4/6 complexes are formed and phosphorylate the retinoblastoma protein (Rb). Phosphorylation of Rb results in the release of the transcription factor E2F, thus enabling transcription of early E2F response genes. In late G1, Rb is further phosphorylated by cyclin E–Cdk2, allowing further transcription of E2F responsive genes and passage through the G1/S restriction point. In addition, Cks1B facilitates the p27 degradation by forming a bridge between Cdk2-cyclinE–p27 and Skp2. Once the cells are in S phase, cyclin A–Cdk2 complexes are formed. At the end of the interphase, cyclin A–Cdk1 is activated to initiate the mitosis and the progression through the mitosis depends on cyclin B–Cdk1. Importantly, the function of these cyclin-Cdk dimers can be modulated by either activators (Cdc25A and Cdc25C) or inhibitors (INK4 family and Kip/Cip family of Cdk inhibitors). In early mitosis, aurora A kinase (AurA) and polo-like kinase 1 (Plk1) promote the activation of cyclin B–Cdk1 complexes. In addition, both Plk1 and AurA can, directly or indirectly, stimulate the activator Cdc25C. During prometaphase the spindle assembly checkpoint (SAC) pathway is blocking the activation of the anaphase-promoting complex/cyclosome (APC/C). However, once bi-orientation is achieved the APC/C is activated by Cdc20 allowing degradation of cyclin B. The formation of APC/C-Cdc20 is also stimulated by Plk1. In late mitosis, APC/C is activated by Cdh1 and important mitotic proteins are degraded. P: prophase, PM: prometaphase, M: metaphase, A: anaphase and T: telophase.

The cell cycle is a very precise process that is regulated by the coordinated activation of cyclin-dependent kinases (Cdk) and several checkpoint pathways, which ensures proper transmission of the genome into the daughter cells (Figure 2) [21–23]. The function of Cdks is regulated by cyclins and cyclin-dependent kinase inhibitors (CKI). Interaction between cyclins and Cdks results in the activation of the kinases. Since cyclins are synthesized and destroyed at specific time points in the cell cycle, they periodically regulate the kinase activity. On the other hand, CKI block the function of Cdks. Two families of CKIs are identified: the INK4 family (p15 [INK4b], p16 [INK4a], p18 [INK4c] and p19 [INK4d]), which bind to Cdk4 and Cdk6 to prevent interaction with cyclin D and the Cip/Kip family (p21 [Cip1], p27 [Cip2] and p57 [Kip2]), which bind almost all cyclin–Cdk complexes [19, 21].

Progression through the interphase depends on three Cdks (Cdk4, Cdk6 and Cdk2) and their binding partners (cyclin D, E and A) [22]. In early G1, cyclin D interacts with Cdk4 and Cdk6. These activated cyclin D–Cdk4/6 complexes phosphorylate the retinoblastoma protein (Rb). Unphosphorylated Rb blocks the transcription factor E2F. Phosphorylation of the Rb by cyclin D–Cdk4/6 initiates the release of E2F and transcription of early E2F responsive genes, such as cyclin E and cyclin A. Cyclin E can activate Cdk2 in late G1, which completes the phosphorylation of Rb and leads to further transcription of E2F responsive genes and eventually G1/S transition. Passage through the Rb/E2F-controlled restriction point results in the initiation of DNA replication [24–26]. Cyclin A activates Cdk2 in the S phase. This complex phosphorylates proteins involved in DNA replication, such as DNA polymerase α [17, 27]. At the end of the interphase, the master mitotic kinase Cdk1 is activated by cyclin A to initiate the prophase. Cyclin A is degraded after the nuclear envelope breaks down and cyclin B–Cdk1 complexes are formed. This complex is responsible for progression through mitosis [22]. Once all chromosomes are aligned in metaphase, Cdk1 activity is blocked by proteasomal degradation of cyclin B. Cyclin B is targeted by the anaphase-promoting complex/cyclosome (APC/C), an E3 ubiquitin ligase which mediates chromosome segregation. APC/C is activated by its co-activator Cdc20 when all chromosomes are attached to microtubules and alignment at the metaphase plate occurs. The activated APC/C-Cdc20 mediates ubiquitylation of key proteins, such as cyclin B and securin. Degradation of both proteins results in respectively the inactivation of Cdk1 and the activation of separase. The latter is a protease that cleaves the cohesin molecules, which are the connections between sister chromatids. This leads to the segregation of chromosomes and subsequently the anaphase onset [20, 28, 29]. Thereafter, there is a switch from APC/C activated by Cdc20 to APC/C activated by Cdh1 (APC/C-Cdh1). This complex mediates the ubiquitylation of several DNA replication (geminin, Cdc6 and Skp2) and mitotic (cyclin A/B, polo-like kinase 1, aurora kinases A/B and Cdc20) proteins and the mitotic exit. In late mitosis, inactivation of Cdk1 causes chromosome decondensation, reformation of the nuclear envelope and cytokinesis [28, 29]. Besides the Cdks, other mitotic kinases also help controlling cell division such as polo-like kinases (Plk) and aurora kinases [20]. Plk1 promotes the activation of cyclin B1 and Cdc25C (an activator of Cdk1) and subsequently triggers mitotic entry. Moreover, Plk1 is also involved in maturation of centrosomes, spindle assembly, spindle checkpoint and exit from mitosis. Expression levels of Plk1 fluctuate during the cell cycle and are the highest at G2/M transition [30, 31]. Aurora kinases are also involved in the maturation of centrosomes, spindle assembly, chromosome segregation and mitotic exit. Aurora A kinase promotes mitotic entry by phosphorylating Plk1 in the G2 phase and activating cyclin B–Cdk1 complexes. On the other hand, aurora B kinase regulates the spindle checkpoint and cytokinesis [32, 33]. In addition, the cyclin-dependent kinase subunit 1B (Cks1B) also has a function in the cell cycle by interacting with Cdk2. Cks1B plays a role in the S-phase kinase associate protein 2 (Skp2)-mediated p27 ubiquitylation by interacting with both Skp2 and the cyclin E–Cdk2–p27 complex. Cks1B facilitates p27 ubiquitylation and degradation, hence the G1/S transition is promoted (Figure 2). In addition, Cks1B might also regulate Cdk1 activation, by functioning as the link between the cyclin B–Cdk1 complex and either Wee1/Myt1 kinases (inactivator of Cdk1) or Cdc25 phosphatase (activator of Cdk1). Other functions of Cks1B in cell cycle involve the degradation of cyclin B and cyclin A [34, 35].

### Cell cycle checkpoints

Several checkpoint pathways are described throughout the cell cycle (Figure 2). The DNA damage response pathways in interphase prevent proliferation until the DNA is properly repaired and the checkpoint pathways in mitosis ensure correct segregation of chromosomes [17]. DNA damage at the G1/S transition is sensed by the ataxia telangiectasia mutated (ATM) kinase, which phosphorylates the tumor suppressor protein p53. This leads to stabilization and accumulation of p53 and subsequently the induction of p21. Inhibition of cyclin E–Cdk2 complexes by p21 results in the inability to pass the Rb/E2F-controlled restriction point and causes a sustained G1 arrest. In addition, there is a p53-independent mechanism providing a rapid and transient delay in G1. The ATM kinase can also phosphorylate checkpoint kinase 2 (Chk2), resulting in the phosphorylation of Cdc25A (an activator of cyclin E/A–Cdk2). Subsequently, ubiquitin-dependent proteolysis of Cdc25A occurs, leading to inhibition of cyclin E–Cdk2 complexes. After proper DNA repair the checkpoint pathway becomes inactive and cells can enter the S phase [17, 23, 26]. DNA damage in S phase and at the G2/M transition activates the ataxia telangiectasia and Rad3-related (ATR) kinase and subsequently checkpoint kinase 1 (Chk1). Chk1 mediates the degradation of Cdc25A and Cdc25C, which results in inhibition of respectively cyclin A–Cdk2 in S phase and Cdk1 at G1/M transition [17, 23, 27]. The safeguard mechanism in mitosis that prevents errors in chromosome segregation is the spindle assembly checkpoint (SAC) pathway. The progression into anaphase is delayed by the SAC pathway until all chromosomes are properly attached to the mitotic spindle and it involves the mitotic checkpoint complex (MCC). This complex is located at the kinetochores, an important part of the chromosome for spindle attachment. Unattached kinetochores cause the formation of the MCC, which includes BubR1, Bub3 and Mad2 proteins associated with Cdc20. The MCC inhibits Cdc20 and subsequently the APC/C activity. The target proteins of the APC/C-Cdc20, namely securin and cyclin B, are not degraded and the cells stay in metaphase. The SAC is switched off when bipolar attachment of chromosomes occurs, resulting in metaphase–anaphase transition [17, 28, 29].

## DEREGULATION OF THE CELL CYCLE IN MULTIPLE MYELOMA

Cell cycle defects are common features in human cancer cells. These defects include unscheduled proliferation, genomic instability (GIN, increased DNA mutations and chromosomal aberrations) and chromosomal instability (CIN, changes in chromosome number) and are causing malignant transformation [36]. Unscheduled proliferation is caused by deregulation of the Cdk activity, which can be the result of mutations in Cdk or their regulators. The main alterations in proteins involved in Cdk activity in cancer are overexpression of cyclin D, cyclin E and Cdk4/6, inactivation of INK4 family members, silencing of p21 and p27 and loss of Rb protein [36, 37]. GIN is the result of alterations in the DNA damage response pathways, causing cell cycle progression in the presence of DNA damage. Mutations in DNA damage response proteins, such as ATM, CHK1/2 and p53 lead to a reduced activation of p21 and subsequently hyperactivation of Cdks. In addition, constitutive activation of Cdc25 phosphatases also causes hyperactivation of Cdks [36]. Aneuploidy and other chromosomal alterations (CIN) are caused by mutations in proteins involved in chromosome separation during mitosis. As mentioned before, proper chromosome separation is controlled by the SAC pathway. Several SAC proteins are frequently mutated in human cancer, such as Bub1, BubR1 and Mad2. Additional proteins have also been implicated in CIN, including Plk1 and aurora kinase A/B, Nek2, Cdc20, Cdc25, cyclins A/B/E and Cdk1 [36, 37]. Below we provide an overview of genes and proteins involved in cell cycle deregulation in myeloma cells.

### Unscheduled proliferation of myeloma cells

**Figure 3:**
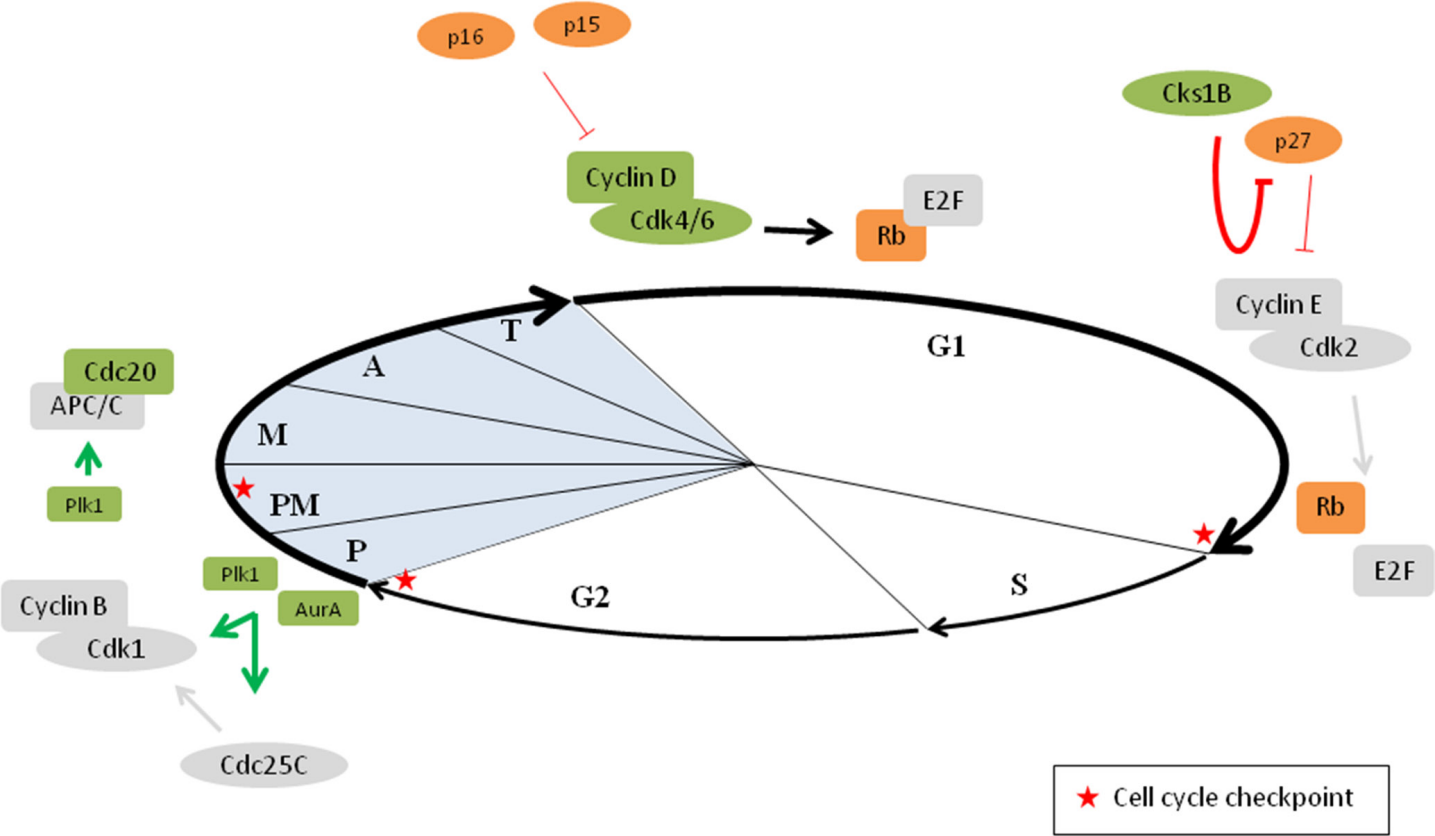
Deregulation of the cell cycle in multiple myeloma Defects in cell cycle progression is a common feature in cancer cells. In multiple myeloma, cyclin D overexpression is observed in virtually all MGUS and MM patients. In addition, overexpression of Cdk4/6, loss of the INK4 family inhibitors and loss of Rb protein is also frequently observed. Together these events result in more E2F release and hence progression through the G1/S restriction point. Cks1B overexpression is inversely linked to p27 expression, which promotes the G1/S transition. In addition, Aurora A kinase (AurA) and polo-like kinase 1 (Plk1) levels are also frequently increased in MM cells, resulting in increased activation of cyclin B–Cdk1 complexes. Finally, Plk1 and Cdc20 proteins are also overexpressed in MM cells leading to more APC/C activation. Proteins depicted in orange are decreased in MM, proteins depicted in green are increased in MM, P: prophase, PM: prometaphase, M: metaphase, A: anaphase and T: telophase.

The unscheduled proliferation of myeloma cells is mainly caused by deregulation of cyclin D and the INK4 family inhibitors (Figure 3). Deregulation of cyclin D expression is one of the key hallmarks of MM and is most often caused by IgH locus translocations. As mentioned earlier, the most frequent translocation is t(11;14), which results in an upregulation of cyclin D1 expression. Both t(14;16) and t(14;20) translocations result in an overexpression of a MAF transcription factor (respectively c-MAF and MafB) and t(4;14) translocation results in the deregulation of MMSET. Overexpression of MAF family members and deregulation of MMSET subsequently lead to upregulation of cyclin D2. Upregulation of cyclin D3 expression is associated with the t(6;14) translocation. Thus, all these alterations result in the deregulation of cyclin D expression and eventually deregulation of the G1/S transition [38–40]. In addition, increased levels of cyclin D1 or cyclin D2 also occur in hyperdiploid MM [38, 41]. Most of the hyperdiploid tumors bi-allelically express cyclin D1. This could be caused by the trisomic chromosome 11, which harbours the cyclin D1 gene [42]. Another mechanism of cyclin D1 and cyclin D2 upregulation might be the downregulation of specific micro RNAs (miRNA) in hyperdiploid tumors, including miR-425, miR-152 and miR24. This downregulation causes the upregulation of cyclin D1, FGFR3, MafB and TACC3 and subsequently an increase in both cyclin D1 and cyclin D2 [43]. Based on the spiked expression of genes deregulated by primary IgH translocations and the universal overexpression of cyclin D genes, 8 TC (translocation/cyclin D) groups have been identified in myeloma patients. Half of the groups, namely the 11q13, 6p21, 4p16 and Maf group are based on the recurrent translocations in myeloma, while the remaining half is based on the increased expression of cyclin D1 and/or cyclin D2 (D1, D1+D2, D2 and none group). The none group shows no increased levels of any of the cyclin D genes [38, 41, 42]. Importantly, all translocations leading to cyclin D2 upregulation, namely t(4;14), t(14;16) and t(14;20) are associated with a poor prognosis in myeloma patients. In contrast, translocations related to the upregulation of cyclin D1 and cyclin D3 are considered to have neutral prognostic implications and hyperdiploidy is associated with a more favourable survival. Among these hyperdiploid MM patients, trisomy 3 and 5 are associated with a better overall survival and trisomy 21 with a poor outcome [39]. More recently, whole exome sequencing and whole genome sequencing of MM patient samples also identified mutations in cyclin D1 that are associated with a negative impact on survival [44, 45].

Thus, it is clear that deregulation of a cyclin D gene is a unifying, early oncogenic event in MGUS and MM. However, aberrant expression of cyclin D alone is rarely associated with increased proliferation. Phosphorylation and inactivation of the Rb protein by cyclin D–Cdk4/6 is the key to cell cycle progression (Figure 3). In addition to cyclin D overexpression, increased Cdk4 and Cdk6 are also described in myeloma. Specific miRNAs such as miR-29b and miR-34, which normally regulate the Cdk4/6 levels, are downregulated in myeloma resulting in this increased Cdk4/6 expression [46]. The mutually exclusive cyclin D1–Cdk4 and cyclin D2–Cdk4/6 pairing is able to inactivate the Rb protein, even in the presence of CKIs. High Cdk6 expression is associated with a poor prognosis in myeloma patients, but no association was seen with Cdk4 expression [47]. Importantly, it has also been described that Myc oncogene deregulates the cell cycle by inducing the expression of several G1 phase cyclins and Cdk (including cyclin D, cyclin E, Cdk2, Cdk4 and Cdk6) [48]. During myeloma progression, increased Myc expression is observed in 15% of the newly diagnosed and 50% of the advanced myeloma patients. This increase is caused by complex translocations involving Myc gene and is associated with a poor prognosis in myeloma patients [39, 42, 48].

Loss of INK4 family inhibitors also plays a role in myeloma pathogenesis. Inactivation of p16 and p15 genes occurs frequently in myeloma cases, due to hypermethylation and/or deletions. Inactivation of p16 and p15 contributes to cell proliferation by the loss of Cdk4/6 inhibition and subsequently phosphorylation of Rb. Alterations in both p16 and p15 are reported in respectively more than 50% and 67% of the myeloma patients [49]. However, neither p16 nor p15 methylation is associated with survival in myeloma patients [49, 50]. In addition, p18 deletions are found infrequent in myeloma [51–53]. In contrast to hypermethylation of the INK4 family genes, methylation of the Cip/Kip family genes is infrequent or absent in myeloma patients. Both p21 and p27 methylation are absent and p57 methylation occurs infrequent in MM patients [54]. However, as mentioned earlier, Cks1B is also an important regulator of p27 and is frequently overexpressed in MM (Figure 3). Cks1B gene amplification is associated with the gain of chromosome 1q, which is one of the most common genetic abnormalities in MM [55–62]. The Cks1B overexpression is inversely linked to p27 expression and is associated with a significantly shorter survival [55–61]. Moreover, Cks1B is one of the genes included in the high risk score designed by Shaughnessy et al. [62].

Finally, the Rb gene can also be deleted in myeloma. The Rb gene is located at chromosome 13, and the 13q deletion causes deletion of the Rb gene. However, most of these deletions are hemizygous and thus not associated with loss of Rb protein. Moreover, loss of Rb protein is only detected in 10% of the myeloma cases and is not associated with poor prognosis in myeloma patients [39, 52, 63]. Besides the 13q deletion, mutations in Rb gene are also described in myeloma. These mutations also appear to be prognostically neutral [44, 45]. Consequently, Rb is considered to be no major target in MM.

### Genomic instability of myeloma cells

Alterations in genes involved in the DNA damage response (such as ATM, ATR, Chk1/2 and p53) result in cell cycle progression in the presence of DNA damage and eventually genomic instability of myeloma cells [39]. ATM and ATR alterations (mutations and deletions) occur in a small subset of myeloma patients, respectively 4.3% and 1.5% of newly diagnosed patients [45, 64]. Both ATM and ATR mutations are associated with a trend toward an impaired prognosis [45]. In addition, p53 alterations are also detected in MM. The p53 gene is located at chromosome 17 and deletions in this chromosome (17p) are associated with p53 deletions. Besides the 17p deletion, mutations (TP53) can also occur in the p53 gene. Both alterations are rarely detected in newly diagnosed patients (17p del: 9.5% and TP53 mutations: 3%), however the incidence increases in later stages of the disease. Moreover, p53 alterations are significantly associated with a poor survival of myeloma patients [45, 65, 66]. Importantly, combined ATM, ATR and TP53 alterations are present in 14.5% of the myeloma patients and have a significant negative impact on overall survival [45]. Moreover, p53 is negatively regulated by MDM2 and the overexpression of MDM2 causes proliferation and survival of myeloma cells [67, 68]. MDM2 gene is located at chromosome 12 and the elevated MDM2 expression in myeloma could be a result of chromosome 12 diploidy (8%) or trisomy (8%) [69]. Another mechanism for the overexpression of MDM2 in myeloma is the epigenetic silencing of miR-192, miR-194 and miR-215 [67]. Other substrates of ATR-Chk1 and ATM-Chk2, besides p53 and MDM2, are Cdc25, BRCA1, BRCA2, FOXM1, E2F1, Wee1 and Rad51 [70]. So far, there is no definitive proof that neither Chks nor most of their substrates are deregulated in myeloma. However, it has been reported that BRCA1, BRCA2 and Rad51 expression levels are increased in melphalan-resistant myeloma cell lines. Furthermore, the Fanconi Anemia (FA)/BRCA pathway contributes to acquired melphalan resistance and inhibition of this pathway may prevent the acquired resistance in myeloma cells [71, 72].

Only very recently, yet another protein family involved in the DNA damage response, namely the RECQ helicases, was reported to be involved in MM pathophysiology. These proteins are DNA unwinding enzymes involved in homologous recombination, repair of damaged DNA and DNA damage checkpoints. In MM, RECQ1 was found to be significantly overexpressed and associated with poor prognosis in patients. Moreover, this RECQ1 overexpression protected the MM cells from cytotoxicity mediated by the standard of care agents melphalan and bortezomib [73, 74].

### Chromosomal instability of myeloma cells

In cancer cells, the increased mutational rate of oncogenes or tumor suppressor genes induced by CIN can provide a proliferative advantage. On the other hand, a too strong or acute CIN could have an adverse effect on cancer cells. The adverse effect is either cell cycle arrest or cell death [75]. Prevention of CIN depends on both the mitotic spindle and the centrosomes, which are responsible for the integrity of the chromosomal content [76]. Myeloma is characterized by high chromosomal instability and aneuploidy, suggesting a disruption of cell cycle checkpoints in myeloma. Deregulation of SAC and kinetochore components are associated with missegregation of chromosomes. The expression of Bub1β, a kinase involved in the SAC pathway, is significantly increased in patients with aggressive myeloma. This increased Bub1β expression promotes myeloma cell proliferation and is associated with a poor survival rate. The induced proliferation occurs through the APC/C-Cdc20–cyclin B1 pathway, which is important for the chromosome separation at the metaphase–anaphase transition [76–78]. Moreover, gene expression analysis of newly diagnosed myeloma patients revealed a higher Cdc20 expression in high-risk patients, correlating with poor prognosis [79]. Next to Cdks, there are three other mitotic kinase families and deregulation of these kinases also contribute to chromosome aberrations [76]. The first mitotic kinase family are the aurora kinases (A, B and C). These kinases have a role in spindle formation, activation of cyclin B–Cdk1 as well as cytokinesis. Increased aurora A kinase expression in myeloma has been associated with centrosome amplification (Figure 3), which is characterized by combinations of abnormal structure and function, and increase in number and size and can contribute to CIN [76, 80]. This amplification is present in about one third of the myeloma patients [81]. However, the prognostic value of centrosome amplification in myeloma is contradicting. The previously used centrosome index (based on gene expression) demonstrates an association with poor prognosis in myeloma patients. In contrast, a more recent study (using immunofluorescent staining) indicates a better prognosis in newly diagnosed myeloma patients who are centrosome amplification positive [82]. The second mitotic kinase family are the polo-like kinases (1, 2, 3, 4 and 5). They all play a role during different parts of mitosis such as mitotic entry, spindle formation, mitotic exit and cytokinesis. In myeloma, Plk1 is increased and may induce chromosome missegregation (Figure 3). Moreover, this Plk1 overexpression is associated with a poor prognosis [76, 80, 83]. The third mitotic kinase family is the NIMA-related protein kinases (Neks). Nek2 plays a role in spindle formation and chromosome segregation and its expression is increased in myeloma resulting in aneuploidy and CIN. Mad2 and Cdc20 (both involved in metaphase–anaphase transition) directly bind to Nek2 and an increase in Nek2 results in the upregulation of Mad2 and Cdc20. Moreover, a correlation between Nek2 expression and drug resistance, relapse and poor outcome is also reported in myeloma [84].

## TARGETING THE CELL CYCLE IN MM

Deregulation of genes and/or proteins involved in cell cycle regulation, plays an important role in cancer pathogenesis in general and more specific in MM pathogenesis. Moreover, proliferation of myeloma cells as assessed by the gene expression-based proliferation index, is a central, independent prognostic factor [85]. Therefore, targeting these cell cycle regulators represents a promising approach in myeloma treatment. Below we discuss the most promising strategies targeting the cell cycle in myeloma.

### Cdk inhibitors

From the above it is clear that cyclin D deregulation is a key hallmark in MM. Moreover, alterations in the cyclin D–Cdk4/6–Rb–INK4 pathway are also frequently observed. Therefore, Cdks represent attractive therapeutic targets in myeloma [86]. In general, inhibition of the interphase Cdks (targeting cell cycle entry) induces a cell cycle arrest or quiescence instead of apoptosis. In contrast, inhibition of Cdk1 (targeting mitotic entry) results in a cell cycle arrest followed by either apoptosis or mitotic slippage. The latter can lead to either an arrest in the next interphase, progression through the cell cycle or cell death. However, Cdk1 is essential for different processes in the cell cycle and it is likely that a strong inhibition of Cdk1 induces toxicity in normal cells, thus preventing the therapeutic use of Cdk1 inhibitors [75]. Inhibition of other Cdks, such as the transcriptional Cdks Cdk7/9 prevents the phosphorylation of the carboxy-terminal domain of RNA polymerase II, resulting in decreased transcription of transcripts with short half-lives (including genes encoding for anti-apoptotic family members, cell cycle regulators, ...). This will lead to a decline in cellular levels of these proteins and subsequently may induce apoptosis [87].

### Pan-Cdk inhibitors

Several pan-Cdk inhibitors have been investigated in myeloma. First generation pan-Cdk inhibitors are flavopiridol and seliciclib (Roscovitine or CYC202) [86, 88]. Flavopiridol targets several kinases including Cdk1, Cdk2, Cdk4, Cdk6 and Cdk7 [86]. In contrast, seliciclib is slightly more specific by targeting Cdk2, Cdk7 and Cdk9. Both compete with ATP for the binding site on Cdks thereby blocking the formation of the activated kinase complex. Flavopiridol and seliciclib induce apoptosis in myeloma cell lines and primary cells and this induced apoptosis is associated with Mcl-1 downregulation [88, 89]. Flavopiridol was also shown to synergistically enhance the anti-myeloma effect of Bcl-2 antagonists, bortezomib and TRAIL, while seliciclib potentiates the anti-myeloma activity of doxorubicin and bortezomib [88, 90–92]. Of interest, the sensitivity of myeloma cells to seliciclib was reported to be associated with cyclin E1 expression; with high cyclin E1 expression correlating with a low sensitivity [93]. Moreover, low p18 expression is also associated with a better response to seliciclib in myeloma cells [94].

Next generation pan-Cdk inhibitors are SNS-032, AT7519, dinaciclib (MK7965 or SCH727965), TG02, RGB-286638, LCQ195 and sangivamycin-like molecule 6 (SLM6) amongst others. All these next generation pan-Cdk inhibitors inhibit both Cdk2 and Cdk9 and most of them also inhibit Cdk1, Cdk5 and/or Cdk7. In addition, AT7519, RGB-286638 and LCQ195 are also able to inhibit Cdk3, Cdk4 and/or Cdk6. Similar to flavopiridol and seliciclib, these pan-Cdk inhibitors induce cell death and reduce transcription efficiency in myeloma cells [95–102]. For SNS-032 and TG02 induced apoptosis is also associated with Mcl-1 downregulation [95, 98], while AT7519 and dinaciclib mediated apoptosis seems associated with dephosphorylation of the glycogen synthesis regulator glycogen synthase kinase 3b (GSK-3b) and disruption of the inositol-requiring enzyme-1 (IRE-1) arm of the unfolded protein response (UPR) respectively [96, 97]. Importantly, both TG02 and LCQ195 are able to overcome the protective effects of BM stromal cells, IL6 and IGF1. In addition, Cdk inhibition by TG02 enhances the anti-myeloma effect of the standard of care agents bortezomib and lenalidomide. Moreover, bortezomib-treated patients showed a significant shorter survival when a high expression of a cluster of genes suppressed by LCQ195 is observed [98, 99, 101]. Finally, AT7519, dinaciclib, TG02, RGB-286638 and SLM6 were all shown to reduce tumor growth of human myeloma cells in xenograft mice [96, 97, 99, 100, 102].

### Inhibition of Cdk4/6

Although pan-Cdk inhibitors have proven their anti-myeloma activity, selective Cdk4/6 inhibitors seem to be more attractive agents due to the important role of Cdk4/6 in regulating MM cell cycle progression and the toxic effects observed when targeting other Cdks, such as myelosuppression and enteropathy [103]. Palbociclib (PD0332991) selectively inhibits Cdk4/6, causing a G1 arrest in primary myeloma cells. Palbociclib does not induce apoptosis by itself, however in combination with dexamethasone or bortezomib myeloma cell death was substantially enhanced [104, 105]. The anti-myeloma activity of palbociclib was also validated in 2 different mouse models (MM1.S xenograft and 5T33MM model) and these studies demonstrated that palbociclib sensitizes the tumor cells to bortezomib killing [105, 106]. Another Cdk4/6 inhibitor, namely P276-00, also blocks the binding between cyclin D1 and Cdk4 by competing with ATP for the ATP-binding site on Cdk4. In myeloma, P276-00 induces a cell cycle arrest or caspase-dependent apoptosis, preceded by inhibition of Rb phosphorylation. Moreover, P276-00 overcomes the survival and drug resistance signals provided by the BM niche and sensitizes the MM cells to bortezomib [107]. The anti-myeloma effect of P276-00 was also validated in RPMI-8226 and MM1.S xenograft mice. Of interest, P276-00 was also reported to target Cdk9. Thus, it is likely that the induced apoptosis is also the result of blocking transcription [107, 108].

### Microtubule targeting agents

Microtubules are involved in the migration of the chromosomes during mitosis. Microtubules interact with the kinetochores on the chromosomes and when bi-orientation of chromosomes is achieved the mitotic checkpoint (SAC) is turned off. MTAs can be divided in microtubule stabilizing agents that enhance the polymerization of microtubules and destabilizing agents that inhibit polymerization of microtubules. Thus, these agents disrupt the normal microtubule dynamics and lead to an impaired formation of the spindle, chromosome alignment and SAC activation. The latter will prevent APC/C-Cdc20 activation and subsequently cause a cell cycle arrest, resulting either in cell death or in mitotic slippage [75, 109].

By far the most tested and widely used MTA in myeloma therapy is vincristine. However, the use of vincristine in myeloma patients is associated with multidrug resistance development and vincristine should therefore be replaced [110]. Despite the promising preclinical effects of other MTAs from the same generation (paclitaxel and vinblastine) and from the next generation (docetaxel and vinorelbine), they showed little or no anti-myeloma activity while inducing severe side effects [111–114]. More recent developed agents with microtubule targeting activities are the isocourmarin derivate 185322, the thalidomide analogue 5HPP-33, CYT997 and PBOX-15 (pyrrol-1,5-benzoxazepine-15). All these agents function as microtubule destabilizing agents. Treatment with these agents results in a M phase arrest and induction of apoptosis in both myeloma cell lines and primary cells [110, 115–117]. Apoptosis induced by 185322 and 5HPP-33 is caspase 3-mediated and for 185322 the induced apoptosis is also associated with phosphorylation of Bcl-2 [115, 116]. Moreover, for CYT997 synergistic *in vitro* and *in vivo* anti-myeloma effects were observed when this agent was combined with bortezomib [117]. Finally, PBOX-15 treatment has been shown to increase DR5 expression and consequently potentiate TRAIL-induced apoptosis [110].

### Motor protein targeting agents

Kinesin motor proteins, such as Eg5 are key regulators of the mitotic spindle. Eg5 is involved in both centrosome separation and bipolar spindle formation and inhibition results in monopolar spindles and a SAC-dependent mitotic arrest [75, 109]. In general, spindle poisons result in a cell cycle arrest that eventually might end in cell death or mitotic slippage [75].

Eg5 inhibitors tested so far in myeloma include BRD9875 and filanesib. BRD9876 is selective for microtubule bound Eg5 and inhibits myeloma cell growth and causes a rapid arrest in G2/M phase. Furthermore, BRD9876 can overcome the proliferative effect of BM stromal cells [118]. Filanesib (ARRY-520) is another, highly selective Eg5 inhibitor. Inhibition of Eg5 by filanesib causes an aberrant mitotic arrest and apoptosis in Mcl-1 dependent myeloma cell lines that are able to degrade Mcl-1 during mitotic arrest [119]. Moreover, filanesib has been shown to synergize with pomalidomide and dexamethasone and this both *in vitro* and *in vivo* in MM1.S xenograft mice [120]. Recently, the anti-myeloma activity of filanesib and melphalan was also investigated. This study showed that the interaction between filanesib and melphalan is dependent on the sequence of treatment. Melphalan administration prior to filanesib causes a S phase arrest and inhibition of filanesib induced apoptosis, whereas filanesib induced apoptosis is enhanced when filanesib is added prior to melphalan [121].

### Aurora kinase inhibitors

The family of aurora kinases consists of 3 members, all involved in either mitosis (aurora A and B kinase) or meiosis (aurora C kinase). The inhibition of both aurora A and B kinase induces cell death, however through different mechanisms. Targeting aurora A kinase induces mitotic spindle assembly defects, which result only in a transient arrest in mitosis. Aurora B kinase inhibition overrides the SAC causing polyploidy [122]. Similarly to MTA, targeting aurora kinases can result either in cell death or mitotic slippage causing tetraploid cells [75].

### Pan-aurora kinase inhibitors

VX-680 acts by inhibiting all aurora kinases. Treatment of myeloma cell lines and primary MM cells with VX-680 results in a cell cycle arrest followed by induction of tetraploidy and apoptosis [80, 123–125]. These effects were reported to be most likely dependent on aurora A kinase inhibition [124]. VX-680 has also been described to overcome the protective effect of IL6, activating mutations of N-Ras and BM stromal cells [80, 125]. Moreover, additive effects were obtained by combining VX-680 with bortezomib, doxorubicin and dexamethasone [123, 125]. More recently, VX-680 treatment was also shown to target the population of cells with tumor-initiating characteristics [126]. In addition, both VX-680 and PHA-680632 (a second pan-aurora kinase inhibitor) abrogated NF-κB activation induced by TRAIL in myeloma cell lines. Consequently, combining pan-aurora kinase inhibitors with TRAIL induced caspase-dependent apoptosis *in vitro* and significantly reduced the tumor growth compared to either compound alone in RPMI-8226/R5 xenograft mice [127]. Of interest, studies with VX-680 in myeloma cells reported the correlation between receptor for hyaluronan-mediated motility (RHAMM) expression and the extent of centrosome amplification. Therefore, it is suggested that aurora kinase inhibitors could be especially efficient in myeloma patients with an increased RHAMM expression [80, 123]. ENMD-2076 is another inhibitor that targets both aurora kinases and multiple receptor tyrosine kinases. In MM, ENMD-2076 showed significant cytotoxicity against MM cell lines and primary cells. At early time points, ENMD-2076 was reported to inhibit the PI3K/Akt pathway and downregulate survivin and XIAP, while at later time points ENMD-2076 was shown to inhibit aurora kinases and induce a G2/M cell cycle arrest [128]. Furthermore, ENMD-2076 treatment dose-dependently decreased tumor growth in NCI-H929 and OPM-2 xenograft mice [128, 129]. AT9283 is also a multi-target inhibitor with potent activity against all aurora kinases and janus kinases. In myeloma cells, AT9283 treatment inhibited proliferation and induced apoptosis. This induced apoptosis seems to be due to inhibition of both aurora A and B kinase as evidenced by an increase in the number of tetraploid cells and a decrease in phophorylation of histon H3, aurora A kinase and STAT3. In addition, AT9283 synergistically enhanced the anti-myeloma activity of lenalidomide and inhibited tumor growth in MM1.S xenograft mice [130].

### Aurora A kinase inhibitors

Alisertib (MLN8237) is a selective aurora A kinase inhibitor. Alisertib treatment of myeloma cell lines and primary MM cells leads to mitotic spindle abnormalities, G2/M cell cycle arrest and apoptosis. Alisertib upregulates p53 and subsequently p21 and p27. In addition, synergistic anti-myeloma effects are described when combined with bortezomib, doxorubicin or dexamethasone. Finally, *in vivo* treatment of MM1.S xenograft mice significantly reduced tumor growth and prolonged overall survival [131].

### Aurora B kinase inhibitors

Most studies have investigated the anti-myeloma effect of pan-aurora kinase inhibitors and validated aurora A kinase as the main aurora kinase target in myeloma. Nevertheless, the selective aurora B kinase inhibitor AZD1152 showed that this kinase might also be interesting in anti-myeloma therapy. AZD1152 induces apoptotic cell death in myeloma cell lines and primary MM cells and combination with dexamethasone enhances its anti-myeloma effect. In addition, AZD1152 also reduced tumor growth and induced cell death in RPMI-8226 xenograft mice [132].

### Polo-like kinase inhibitors

Polo-like kinases have an important role in the cell cycle by controlling mitotic entry. The Plk family consists of 5 members, of which Plk1 is the most investigated. Plk1 is involved in mitotic entry, coordination of centrosome and cell cycle, regulation of spindle assembly and in cytokinesis [133]. Consequently, inhibition of Plk1 leads to several defects such as mitotic entry delays, defects in centrosome maturation and mitotic spindle abnormalities [134]. As mentioned before, inhibition of mitotic entry and spindle assembly can result in a G2 or prometaphase arrest that eventually ends in apoptosis or the cell cycle arrest can be followed by mitotic slippage resulting subsequently in an arrest in the next interphase, progression through cell cycle or cell death [75]. Indeed, Plk1 inhibition is not only associated with apoptosis, but also with post-mitotic DNA damage and senescence in some human cancer cell lines [134].

The Plk1 inhibitor BI2536 has proven to possess potent anti-myeloma activity. BI2536 treatment results in a G2/M phase arrest and eventually apoptosis in myeloma cell lines and primary cells [83, 135–137]. In addition, synergistic effects were observed for BI2536 combined with 17AAG (HSP90 inhibitor), BI2536 with BEZ235 (PI3K/mTOR inhibitor) and BI2536 together with obatoclax (pan Bcl-2 inhibitor) [137]. Furthermore, in RPMI-8226 xenograft mice BI2536 significantly decreased tumor growth [83]. However, one study demonstrated that the apoptotic effect was decreased both in *in vitro* experiments and in the MM1.S xenograft mouse model of diffuse MM bone lesions where MM cells are in close contact with the BM stromal cells. This raises concerns about the translation to the clinic [135]. A second Plk1 inhibitor that has been tested in MM is the novel agent scytonemin. Scytonemin inhibits myeloma cell growth by inducing a G2/M phase arrest [138].

### Inhibitors of the anaphase promoting complex/cyclosome

Targeting mitotic exit has been suggested to be a better therapeutic approach than targeting either mitotic entry, spindle assembly or mitotic checkpoint, as inhibition of mitotic exit causes a permanent mitotic arrest subsequently leading to mitotic cell death. This is in contrast to the other approaches, which often result in a cell cycle arrest followed by a possible re-entry in a new cell cycle [75]. As mentioned before, the E3 ubiquitin ligase APC/C is a key regulator in mitosis driving mitotic exit. APC/C activity depends on two co-activators, namely, Cdc20 and Cdh1, each controlling different parts of the cell cycle. APC/C-Cdc20 targets both cyclin B and securin for destruction at the end of the metaphase, leading to the start of the anaphase. In contrast, APC/C-Cdh1 is responsible for mitotic exit and maintenance of the early G1 phase [29]. Until now, the main focus in targeting the APC/C has been blocking the APC/C by preventing Cdc20-dependent mitotic progression. This inhibition results in a cell cycle arrest and might eventually lead to cell death [139].

Recently, we showed that Cdc20 is significantly higher expressed in high-risk myeloma patients and this elevated Cdc20 correlates with a poor prognosis [79]. This was later on confirmed by Crawford et al. [140]. In contrast to our observations, Crawford et al. also showed that elevated Cdh1 levels are found in newly diagnosed myeloma patients. An explanation for this contradiction could be the different data sets used in both studies [79, 140]. Nevertheless, we and Crawford et al. both demonstrated that APC/C inhibition using the small molecule inhibitor proTAME induced a cell cycle arrest and caspase-mediated apoptosis in human cell lines and primary MM cells. Moreover, combinations with conventional anti-myeloma drugs such as melphalan, doxorubicin and vincristine resulted in enhanced anti-myeloma effect [79, 140]. These data indicate that the APC/C could be a promising new target for MM therapy. However, *in vivo* validation remains so far impossible due to the fact that proTAME is a prodrug that needs to be activated by esterases to the active, non-cell permeable component TAME. Upon *in vivo* administration, the esterases that are active in the blood stream will process almost all of the proTAME before reaching the target cells, resulting in no uptake of the drug in these cells [141].

### Activation of p53

Activation of p53 results in cell cycle arrest and apoptosis, therefore therapeutic activation of p53 might be an attractive approach in myeloma. Different mechanisms of p53 activation have been reported, such as reactivation by MDM2 inhibition (e.g. Nutlin-3 and RITA) or restoring normal p53 function in p53-mutated cells (e.g. PRIMA-1 and MIRA-1). However, this is beyond the scope of this review. A more detailed overview of p53 targeting in myeloma is described in Herrero A et al. (2016) [142].

## CLINICAL TRIALS IN MM

Given the promising preclinical studies described above, a lot of interest went into evaluating the clinical anti-myeloma activity of agents targeting cell cycle regulators the past years. In relapse/refractory (RR) myeloma patients, different Cdk inhibitors have been or are currently being tested in clinical trials. Despite the promising preclinical results, the use of the first generation pan-Cdk inhibitor flavopiridol as single agent showed limited clinical efficiency in myeloma patients [143, 144]. A phase I study also tested the combination of bortezomib and flavopiridol in RR indolent B cell neoplasms, including MM. Despite the completion of this trial, no results are yet available [145]. Different second generation pan-Cdk inhibitors have also been evaluated in clinical trials. The safety and tolerability of SNS-032 was investigated in chronic lymphocytic leukemia and MM patients. However, due to an early closure of the trial no dose-limiting toxicity and maximum tolerated dose was identified. Moreover, stable disease was observed in only 2 MM patients [146]. In contrast, dinaciclib was recently shown to demonstrate single agent activity in a small group of relapsed myeloma patients (*n* = 27), with 2 patients achieving a VGPR (very good partial response) and 10 patients obtaining stable disease [147]. Based on these promising results, a phase I study is currently testing the side effects and best dose of dinaciclib and bortezomib when given together with dexamethasone in relapsed myeloma patients. In addition, a phase I study testing the safety and efficacy of the PD-1 inhibitor pembrolizumab in combination with dinaciclib is currently actively recruiting [145]. AT7519 is yet another pan-Cdk inhibitor that has recently been tested for its clinical activity either alone or in combination with bortezomib. Although this study has been completed in 2016 no results are published yet [145]. The safety and efficiency of the specific Cdk4/6 inhibitor palbociclib combined with bortezomib and dexamethasone was also evaluated in a phase I/II study in RR myeloma patients. In this study, palbociclib was shown to inhibit Cdk4/6 and hence the cell cycle in most of the patients and the Cdk4/6 inhibition could be reversed by withdrawal of palbociclib. Moreover, combining these 3 agents resulted in an objective response and stable disease in respectively 20% and 44% of the patients [148]. A phase I clinical trial in RR myeloma patients evaluating palbociclib in combination with lenalidomide and dexamethasone was recently terminated due to a low enrolment. Currently, the TAPUR (targeted agent and profiling utilization registry) phase II trial and NCI-MATCH phase II trial are recruiting MM patients with respectively advanced stage and RR disease, with a purpose to investigate the targeted therapy of palbociclib, directed by genetic testing. The TAPUR trial includes patients whose tumor harbours loss in p16 or Cdk4/6 amplifications, while the NCI-MATCH trial includes patients with overexpressed cyclin D [145]. In 2009, a phase I/II study evaluating the safety and efficacy of the specific Cdk4/6 inhibitor P276-00 in MM was withdrawn prior to enrolment. A new phase I/II trial determining the safety of P276-00 was completed in 2012, however, no results have been posted yet [145]. Despite the encouraging clinical results obtained for some of these Cdk inhibitors (especially in combination with standard of care agents), a major drawback of most Cdk inhibitors is their lack of specificity and hence cytotoxicity in clinical use [107]. Indeed, in most of the trials severe (grade 3/4) adverse effects were observed, such as thrombocytopenia and leukopenia [147, 148].

Besides Cdk inhibitors, several mitosis targeting agents are also evaluated in clinical trials including MTA and inhibitors of motor proteins or aurora kinases. As mentioned before, preclinical effects of MTA are promising, however the anti-myeloma activity is limited [110–114]. Despite this limitation a phase II trial is currently recruiting patients to evaluate the safety of paclitaxel in combination with cyclophosphamide and dexamethasone in RR myeloma patients. In addition, the use of the injectable formulation of paclitaxel, named nanoparticle albumin-bound paclitaxel or nab-paclitaxel is also being tested in RR myeloma. The ongoing phase II study aims to determine the efficacy of nab-paclitaxel in these patients. In addition, the safety of nab-paclitaxel in combination with lenalidomide was supposed to be determined in another phase I/II trial. However, this study was terminated early [145]. The more recently developed MTA CYT997 was tested in a phase II trial to determine the single agent anti-myeloma activity in RR myeloma patients. However, the trial was terminated early due to difficulties in enrolling patients [145]. Similar to the Cdk inhibitors, MTAs have been shown to induce severe adverse effects, including neuropathy and neutropenia. In addition, the usage of MTAs has also been associated with the development of multidrug resistance [17, 110].

The efficacy of inhibiting motor proteins is evaluated in MM patients. The main Eg5 motor protein inhibitor tested so far in RR MM patients is filanesib. The safety and efficacy of filanesib in RR MM patients was determined in 2 different phase II studies. One is completed and the other one is still ongoing but no results are available yet [145]. In contrast, one phase I trial showed encouraging anti-myeloma activity when filanesib and bortezomib were administered with or without dexamethasone, with an overall response rate of 20% in all patients and 29% in patients with proteasome inhibitors refractory disease [149]. In addition, the combination of filanesib and carfilzomib in advanced MM was evaluated in a phase II trial. Despite the completion of the study, no results were posted yet [145]. The most common observed adverse effects of filanesib were transient noncumulative neutropenia and thrombocytopenia [149].

Several studies have tested the clinical efficacy of targeting aurora kinases in myeloma patients. A phase I trial evaluated the safety of the selective aurora A kinase inhibitor alisertib in RR patients with haematological malignancies showing a partial response and stable disease in respectively 13% and 28% of these patients. A second trial also evaluated the efficacy of combining alisertib with bortezomib. The overall response rate was 26.9%, with one patient achieving a complete response, 6 patients a partial response and 10 patients stable disease for at least 2 cycles [150, 151]. In addition, 2 pan-aurora kinase inhibitors have been tested in MM, namely ENMD-2076 and AT9283. The safety, tolerability, maximum tolerated dose and clinical benefit of treatment with ENMD-2076 was tested in patients with RR MM. Although this phase I study was completed in 2012 no results are available yet [145]. In contrast, a phase II study recently reported on the efficacy of AT9283 in RR MM. No objective response was observed in any of the 8 patients and the use of AT9283 was associated with toxicity [152]. Both specific aurora A kinase inhibition and pan-aurora kinase inhibition induce side effects, such as neutropenia, thrombocytopenia and leukopenia [150–152]. In Table 1, we provide an overview of trials that are currently ongoing.

**Table 1:** Cell cycle inhibitors in ongoing clinical trials in MM

Name	Target	Development status
**Cdk inhibitors**		
Flavopiridol	Cdk 1,2,4,6 & 7	Phase II
SNS-032	Cdk 2,7 & 9	Phase I
AT7519	Cdk 1,2,4,6,7 & 9	Phase I/II
Dinaciclib	Cdk 1,2,5 & 9	Phase I/II
Palbociclib	Cdk 4/6	Phase I/II
P276-00	Cdk 4–cyclin D	Phase I/II
**Microtubule targeting agents**		
Vincristine	tubulin	FDA approved
Paclitaxel	tubulin	Phase II
CYT997	tubulin	Phase II
**Motor protein targeting agents**		
Filanesib	kinesin protein Eg5	Phase II
**Aurora kinase inhibitors**		
ENMD-2076	Aurora A, B & C kinase	Phase I
AT9283	Aurora A, B & C kinase	Phase II
Alisertib	Aurora A kinase	Phase I

## CONCLUSION AND REMARKS

Cyclin–Cdk dimers are essential regulators of cell cycle progression [21–23]. Deregulation of cyclin D is a key hallmark in MM and alterations in the cyclin D–Cdk4/6–Rb–INK4 pathway occur frequently [38, 86]. Consequently, most of the studies targeting cell cycle in MM have mainly focused on inhibiting Cdk and hence inhibiting cell cycle entry. Based on preclinical data obtained for the Cdk inhibitors, it was suggested that specific Cdk4/6 inhibitors might be more favourable to use in anti-myeloma therapy than pan-Cdk inhibitors, since Cdk4/6 are the main Cdks deregulated in MM patients [103]. In line, the specific Cdk4/6 inhibitor palbociclib was demonstrated to achieve better objective responses in myeloma patients compared to the pan-Cdk inhibitors flavopiridol, SNS-032 or dinaciclib [143, 144, 146–148]. In addition, the only grade 3/4 adverse effects observed in palbociclib treated myeloma patients are thrombocytopenia and neutropenia. Moreover, these adverse effects can be reversed upon withdrawal of the inhibitor [148]. In contrast, the pan-Cdk inhibitors induced much more grade 3/4 adverse effects, such as anemia, neutropenia, leukopenia, diarrhea, thrombocytopenia, pneumonia and fatigue [143, 144, 146, 147]. However, targeting cell cycle entry is known to be associated with quiescence, which could be a possible explanation for the rather limited activity of the Cdk inhibitors in MM [75]. Therefore, other important cell cycle proteins are currently being actively explored as target in anti-myeloma treatment. Preclinical research identified several components of mitosis as possible interesting targets in myeloma treatment, including kinesin motor protein Eg5, aurora A kinase and Plk1, which are all involved in the spindle formation [75, 80, 83]. From the use of MTAs in current cancer treatment, it is known that interference with microtubule formation and the spindle assembly checkpoint results in impaired cancer cell cycle progression and eventually cell death or mitotic slippage [75, 109, 153]. It is however anticipated that the more recently developed spindle inhibitors would target cell cycle progression in a more cancer cell-specific manner, which avoids the neuropathy associated with MTAs [154]. Clinical trials with the aurora kinases inhibitors alisertib and AT9283 in myeloma seem to confirm this, since there is no neuropathy observed upon treatment with alisertib or AT9283 [150–152]. For the Eg5 inhibitor filanesib, however, peripheral neuropathy was still observed in a small percentage of patients. In addition, both filanesib and alisertib show only limited single agents activity in myeloma patients, with stable disease being the best response achieved [149, 150]. In combination with a proteasome inhibitor both agents showed remarkably better objective responses; up to 20% for filanesib and 35% for alisertib in combination with bortezomib [149, 151]. Based on these trials, it can be suggested that the newly developed spindle poisons are not better compared to the already used MTAs. It has been previously speculated that the success of MTAs might be due to the ability to target non-mitotic functions of microtubules [75].

Thus, despite all the promising preclinical results obtained by blocking cell cycle progression in myeloma cells, results from most clinical studies are somewhat disappointing. Recently, it has been suggested that blocking mitosis downstream of the SAC pathway could induce cell death more efficiently than agents that affect the SAC [153]. Downstream targeting is more likely to cause a permanent mitotic arrest subsequently resulting in mitotic cell death. Thus, targeting mitotic exit (e.g. by inhibition of APC/C) seems a better strategy than inhibiting mitotic entry [75, 153]. Based on preclinical data reported on the APC/C inhibitor proTAME, it seems that targeting APC/C could indeed be an efficient strategy in myeloma treatment, especially in combination with standard of care agents [79, 140]. However, so far, it remains impossible to evaluate proTAME in clinical trials [141]. Therefore, investigation and development of novel selective APC/C inhibitors is urgently needed to improve the field of cell cycle targeting in MM and cancer treatment in general.

Another explanation for the limited activity of the cell cycle targeting agents observed so far might be that most clinical trials did not include predictive markers for the selection of probable responders. By using these markers, a patients cohort could be selected that will benefit from the treatment. In concurrence with this, two phase II trials testing palbociclib in advanced/RR MM (the TAPUR and NCI-MATCH trial) are currently actively recruiting MM patients with an aberrant Cdk4 or cyclin D expression. However, further research is still necessary to identify these predictive markers and find the best suitable marker to use in clinic. Secondly, most trials also neglected to determine the optimal timing and/or sequence of administration. Only very recently, it was shown that the sequence of administration is crucial in combination treatment. In this study, treatment of melphalan prior to filanesib causes a S phase arrest and inhibition of filanesib induced apoptosis, whereas filanesib induced apoptosis is enhanced when filanesib is added prior to melphalan [121]. Finally, in light of the clonal heterogeneity observed in MM patients both at the time of diagnosis and relapse, cell cycle targeting harbours the risks of selecting for the dormant (non-proliferating) and hence resistant clones. Therefore, new combination therapies should be designed in such a way that both the proliferating and dormant cells are targeted at the same time.

In conclusion, MM remains most often an incurable plasma cell disorder and blocking cell cycle progression represents an interesting approach to improve anti-myeloma therapy. However, to enhance the efficiency of cell cycle targeting, additional strategies such as targeting mitotic exit (e.g. APC/C targeting) should be actively explored and novel selective APC/C inhibitors that can be used in preclinical *in vivo* studies and clinical trials should be developed. Moreover, clinical trials should select the patients based on predictive markers to ensure selecting the patient population that is likely to benefit from the treatment.
